# Evaluating long-term antibody responses to booster doses of COVID-19 vaccines in the Pakistani population

**DOI:** 10.12669/pjms.40.2(ICON).8951

**Published:** 2024-01

**Authors:** Shakir Hussain, Fouzia Naseer, Fatima Kanani, Javeria Aijaz

**Affiliations:** 1 Shakir Hussain, Molecular Biology Section, Pathology Department, Indus Hospital & Health Network, Karachi 75190, Pakistan; 2 Fouzia Naseer, Molecular Biology Section, Pathology Department, Indus Hospital & Health Network, Karachi 75190, Pakistan; 3 Fatima Kanani, Chemical Pathology Section, Pathology Department, Indus Hospital & Health Network, Karachi 75190, Pakistan; 4 Javeria Aijaz, Molecular Biology Section, Pathology Department, Indus Hospital & Health Network, Karachi 75190, Pakistan

**Keywords:** BBIBP-CorV, CMIA, COVID-19, Pakistan, pseudotyped, BNT162b2 mRNA, immunoassay, Vaccine

## Abstract

**Background & Objective:**

Nearly 80 million of the Pakistani population received two doses of the BBIBP-CorV vaccine, against SARS-CoV-2, and 2.6 million people received heterologous booster doses up to February 2022. Our objective was to measure the long-term change of antibody titers in persons vaccinated with Pfizer–BioNTech COVID-19 following two doses of BBIBP-CorV.

**Methods:**

Serum specimens from forty-three participants were collected 4-8 weeks following two doses of BBIBP-CorV at the Indus Hospital & Health Network, Karachi. A second set of serum specimens were collected 2-12 months after Pfizer–BioNTech COVID-19 booster dose administration. Chemiluminescent Microparticle Immunoassay (CMIA, Abbott Alinity Quant), and the pseudotyped lentivirus antibody neutralization assay were performed on all specimens. The latter assay was reported as log half-maximal inhibitory concentrations (IC50), calculated using a nonlinear regression algorithm (log [inhibitor] versus normalized response variable slope) in Graph Pad Prism 9. Paired sample t-test was used to ascertain the statistical significance of the difference in means of antibody titers obtained before and after the booster vaccine doses.

**Results:**

Mean log10 values obtained with CMIA before and after the booster dose were 2.90 AU/mL and 3.87 AU/mL respectively, while the corresponding log10 IC50 values obtained through pseudotyped lentivirus antibody neutralization assay were 2.45 and 2.80. These differences were statistically significant with CMIA (p = <0.00001), but not with pseudotyped lentivirus antibody neutralization assay (p = 0.06318.).

**Conclusion:**

A heterologous booster dose with Pfizer–BioNTech COVID-19 vaccine following two doses of BBIBP results in increased total antibody titers, though neutralizing antibody titers may start to wane a few months after the booster dose.

## INTRODUCTION

More than 50 COVID-19 vaccines have been declared safe by WHO.^1^ By now, these vaccines have been administered all over the world, though the coverage is not 100% in all countries. In Pakistan, a total of 333,085,477 COVID-19 vaccine doses have been administered from 3rd February 2021 to 01 March 2023.^2^ About 162.2 million people had received vaccination by Feb 19, 2023. While 73.4% of the population is partially vaccinated.^3^ Specific data on the types of vaccines administered in Pakistan is, however, not publicly available.

The humoral immunity response of the heterologous regimen of BBIBP-CorV with BNT62b2 booster vaccines is reported to be stronger than the response obtained through receiving homologous booster shots of another dose of BBIBP-CorV.^4^ In addition, heterologous booster regimens are safe and well-tolerated. In Pakistan, the Sinopharm COVID-19 vaccine (BBIBP-CorV) for initial vaccination and BNT62b2 as booster have been the more frequently used heterologous vaccine combination.^5^

Following vaccinations, both total and neutralizing antibody titers may rise. Total antibodies (IgM, IgG, or IgA) may not be protective against future infections while neutralizing antibodies are known to be protective.^6^,^7^ Neutralizing antibodies inhibit the binding of the RBD to the ACE2 receptor in cells. It is widely reported that the efficacy of vaccines is correlated with the magnitude of neutralizing antibodies response which these are able to engender.^8^ Different assays are designed to detect both total and neutralizing antibodies. These assays include ELISA-based assays, immunoassays, and live virus assays.^9^ Chemiluminescent immunoassay (CLIA) is one of these assays, which detects total antibodies, while live virus assays are known to be the gold standard for detecting neutralizing antibodies.^10^ Although almost all studies concur that a booster doses can increase the neutralizing antibodies levels and their surrogate markers (e.g., anti-spike IgG) against COVID-19 infection, studies do differ on the reported magnitude of immune response compared to the baseline level before the booster doses. These discrepancies highlight a lack of consensus on findings of the immune response dynamics following the booster dose, especially over a longer period of time.^11^-^13^

Most studies done with the aim of determining post-booster antibody response have been conducted within a timeframe of four weeks following the booster dose.^14^ In this study, we aimed to determine this response beyond two months of booster dose administration, as studies are still needed that can ascertain the long-term magnitude of the immune response to these vaccine combinations. We also wished to ascertain if there is a difference in the magnitude of total antibodies versus neutralizing antibodies after administration of the two-dose and booster combinations used in this study.

## METHODS

All study participants were healthcare workers at the institution who had previously participated in a study to ascertain the immune response following two doses of BBIBP-CorV. Therefore, the baseline (control) data of their immune status after 2 doses of BBIBP-CorV was already existing. For the current study, we approached those study participants who had received a booster dose of BNT62b2, in order to ascertain the change in the antibody response following the booster dose.

### Inclusion criteria

Eligible adult participants having received two doses of BBIBP-CorV and a booster dose of BNT62b2.

### Exclusion criteria

Participants with breakthrough infections were not included in the study. Twelve eligible participants were excluded due to the non-availability of a booster shot date. There were no other exclusion criteria

### Ethical committee approval

The study protocol of this prospective observational study, questionnaires, and consent form were approved by the Institutional Review Board (IRD-IRB#2020_07_018) of Indus Hospital & Health Network (IHHN).

The sampling method was convenience sampling. The questionnaires used for data collection included participant name, age, gender, hospital record ID, history of COVID-19 in the past, vaccination dates, type of vaccines, sample collection date, and date of last positive PCR. Written, informed consent was obtained from all participants before sample collection.

**Fig.1:**
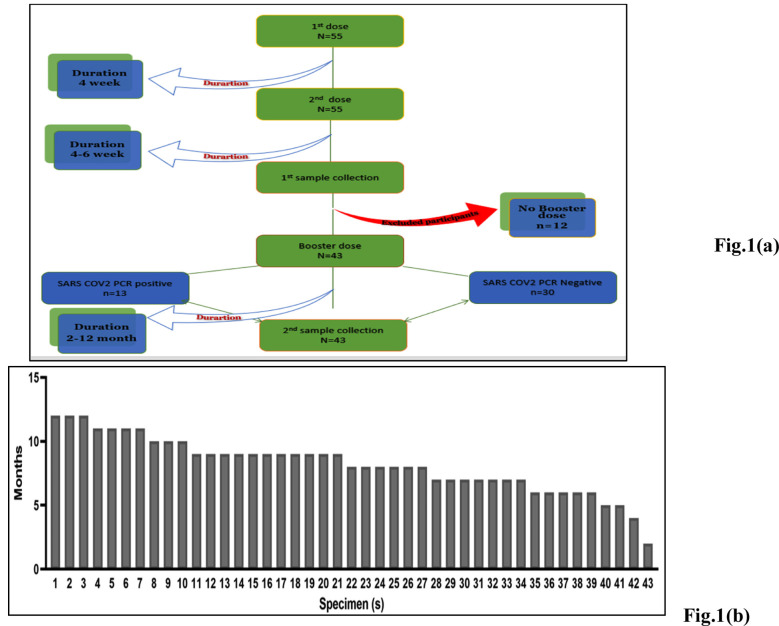
Study design including (a) average duration in the months between the various vaccine doses and sample collection (b) The interval for each study participant in months from first dose to sample collection for the entire study sample.

A total of fifty-five adult participants of both genders were eligible for the prospective observational study and gave informed consent. After excluding the twelve participants with non-availability of booster shot date, forty-three participants fulfilling the inclusion criteria stated above were finally enrolled from April to May 2022 at IHHN, Karachi. Some participants (n=13) had documented exposure to SARS-CoV-2 prior to the two doses of BBIBP-CorV, while others had no known exposure (n=30) to the virus (Fig.1a).

The duration between the booster doses and sample collection varied from 2 to 12 months, with a median duration of 8 months. The duration from the 2^nd^ dose of BBIBP-CorV and the booster dose ranged from 5-12 months (Fig.1b). All study participants had their pre-booster samples collected 4-8 weeks following the second dose of the BBIBP-CorV vaccine, with their pre-booster shot antibody titers determined through the same two assays.

Two tubes of serum samples were collected from each of the forty-three study participants at a variable time interval following the booster shot with BNT62b2. Serum from one of the tubes was used for the SARS-CoV-2 IgG II Quant chemiluminescent microparticle immunoassay (CMIA), according to the manufacturer’s instructions. The assay is designed for the qualitative and quantitative determination of IgG antibodies to SARS-CoV-2 in human serum and plasma. Briefly, the specimen was treated with SARS-CoV-2 antigen-coated paramagnetic microparticles. The SARS-CoV-2 reagent antigen binds to all SARS-CoV-2 IgG antibodies. After washing, an acridinium-labeled anti-human IgG conjugate was added to the mixture. Relative light units (RLU) are used to measure the luminescence that results and are expressed as AU/mL. The amount of RLU found is directly correlated with the total IgG antibodies to SARS-CoV-2.^15^

Serum from the other tube was used for pseudotyped lentivirus antibody neutralization assay following the protocol by Crawford et al, with some modifications.^16^ The lentivirus toolkit was obtained from BEI resources (NR52512, NR52513, NR-52514, 52515, NR-5251, NR-52517, NR-52518, NR-52519, NR-52520). The spike protein sequence comprised that of the Wuhan-Hu-1 variant, with a C-terminal deletion of amino acids 1257-1278, intended to enhance viral titers. Serum specimens were serially diluted and incubated with titrated lentivirus particles prepared using the lentivirus toolkit obtained from BEI resources. The virus-serum mixtures were next added to 293T-ACE2 cells which had been seeded two days prior in flat, clear-bottom, black-walled, poly-L-lysine-treated plates. Assay controls comprised isolated lentivirus particles with cells and cells only. The former represented the maximum possible infection, while the latter represented the maximum possible neutralization. Following incubation for two days, luminescence was detected using Bright-Glo Luciferase Assay System, and a multimode luminescence microplate reader.^15^ Antibody titers obtained with the two assays before and after the booster dose were compared. SARS-CoV-2 IgG II Quant assay (Abbott) results were obtained in AU/mL, while the pseudotyped lentivirus neutralization assay titers were reported as IC50 values obtained through Graph Pad Prism 9. For the pseudotyped lentivirus assay, the average RLU from each specimen dilution was plotted against the corresponding log10 dilution factor of each specimen following normalization. For normalization, virus control represented 100% infection and cell control 0% infection. A nonlinear regression approach (log[inhibitor] vs normalized response variable slope) was used to determine the half-maximal inhibitory doses (IC50) using Graph Pad Prism 9. Graph Pad Prism 9 IC50 values were transformed into a log10 scale. The outcomes of immunoassays (CMIA) were also log10 transformed.

### Statistical analysis

Paired sample t-test was used to compare the baseline antibody titers before the booster dose, and the levels obtained after the booster dose in each sample, both with the CMIA and the pseudotyped lentivirus antibody neutralization assays. The antibody titers before the booster dose (baseline titers) comprised the study controls. To determine the statistical significance of antibody titer differences based on gender and prior COVID-19 infection, t-test was used. Results were deemed statistically significant if the two-sided test’s p-value was less than 0.05.^16^

## RESULTS

**Fig.2:**
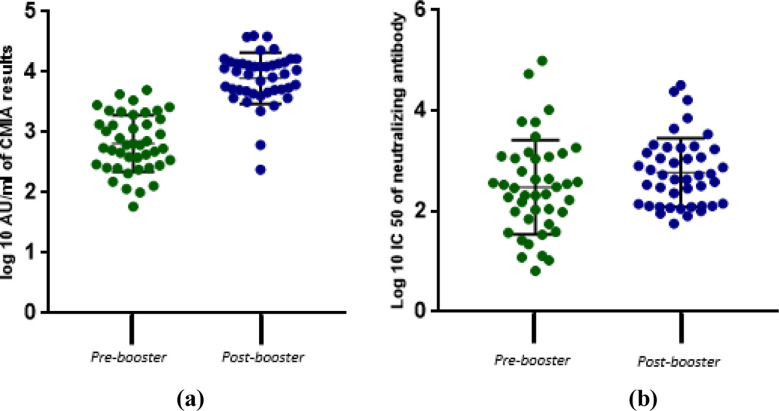
Distribution of antibody titers obtained using CMIA (a) and pseudotyped lentivirus neutralization assay (b) before and after the booster in all study participants. CMIA values are reported as log10 AU/ml values while neutralizing antibodies are reported as log10 IC50. Paired sample t-test was used to determine the difference between the pre-booster i.e. baseline (control) titers, and post-booster antibody titers in both assays. *p=<0.05.

Out of 43 participants, 21 (48.83%) were females, and 22 (51.16%) were males. The mean log10 CMIA values before and after the booster dose were 2.9 AU/mL and 3.87 AU/mL respectively. The mean log10 IC50 values obtained through pseudotyped lentivirus antibody neutralization assay before and after the booster dose were 2.45 and 2.8 respectively. These differences were statistically significant with CMIA (p = <0.00001), but not the pseudotyped lentivirus antibody neutralization assay (p = 0.06318) (Fig.2)

**Figure 3:**
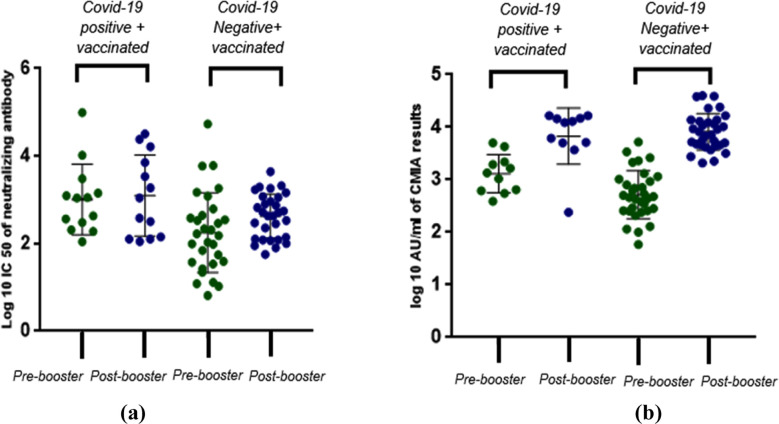
Distribution of antibody titers obtained using CMIA (a) and pseudotyped lentivirus neutralization assay(b) before and after the booster in all study participants segregated by their COVID-19 PCR positivity status. CMIA values are reported as log10 AU/ml values, while neutralizing antibodies are reported as log10 IC50. Paired sample t-test was used to determine the difference between the pre-booster i.e. baseline (control) titers, and post-booster antibody titers in both assays. *p=<0.05

Having observed a substantial rise of antibody titers with the CMIA, but not the pseudotyped lentivirus assay, we then proceeded to analyze if segregating the values by their prior COVID-19 positivity status generated any different results (Fig.3). Using CMIA, the mean log10 titer values obtained before and after the booster dose in COVID-19-positive participants were 3.12 AU/mL and 3.9 AU/mL, respectively. In COVID-19-negative patients, these values were 2.7 AU/mL and 3.87 AU/mL respectively. With pseudotyped lentivirus antibody neutralization assay, the mean log10 IC50 values obtained before and after the booster dose in COVID-19-positive vaccinated individuals were 2.75 and 3.3, respectively. In COVID-19 negative participants, these values were 2.30 and 2.6, respectively. These differences were again statistically significant with the CMIA assay for both sub-groups, but not for the pseudotyped lentivirus assays.

**Figure 4:**
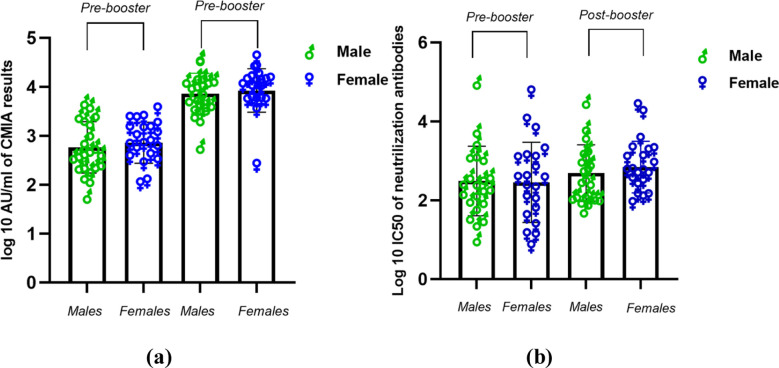
Distribution of antibody titers obtained using CMIA (a) and pseudotyped lentivirus neutralization assay(b) before and after the booster in all study participants segregated by their gender. CMIA values are reported as log10 AU/ml values while neutralizing antibodies are reported as log10 IC50. t-test was used to determine the statistical significance of any differences detected. *p=<0.05

**Figure 5:**
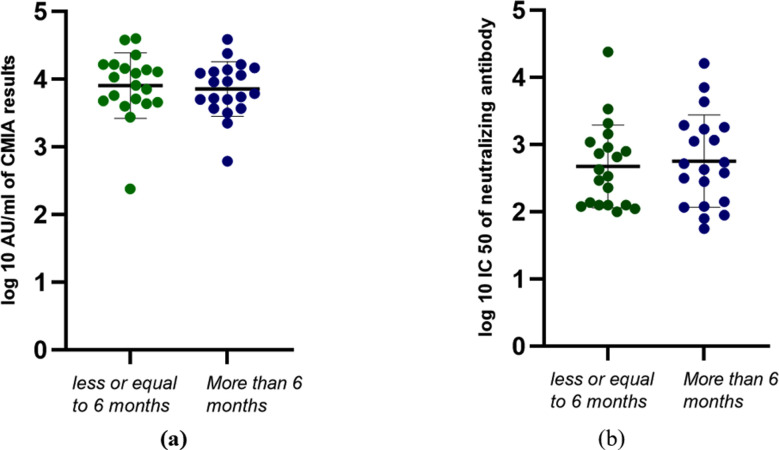
Distribution of antibody titers obtained using CMIA (a) and pseudotyped lentivirus neutralization assay (b) before and after the booster in all study participants segregated by whether they received the booster dose less or more than 6 months following the second dose of BBIBP-CorV. CMIA values are reported as log10 AU/ml values while neutralizing antibodies are reported as log10 IC50. t-test was used to determine the statistical significance of any differences detected. *p=<0.05.

In addition, we did not see any statistically significant gender-based differences in antibody titers following booster doses using either assay (Fig.4). We also divided participants based on whether the interval between booster dose and sample collection was less than 6 months or more than 6 months. We observed no statistically significant reduction of antibody titers more than 6 months following booster dose when compared to titers in samples collected before this 6-month mark (Fig.5).

## DISCUSSION

This study aimed to evaluate the magnitude of change of antibody titers in persons vaccinated with Pfizer–BioNTech (BNT162b2) COVID-19 vaccine as a booster after two doses of BBIBP-CorV. There is a wide reported variance in these titers with titer fold changes before and after the booster dose reportedly ranging from 1.4 to 31. Pérez-Then E, et al. reported 1.4-fold increase in titer after booster dose.^14^ Assawakosri S, et al. reported a 14- to 31-fold increase in titer.^17^ Cheng ZJ, et al. reported a 13.2-fold increase.^18^ Our study shows a rise of titer in the lower range of reported titer increases following the two-dose BBIBP-CorV vaccine and Pfizer booster (BNT162b2). Apart from actual biological differences, another likely source of this variation is differences in methodologies used for the ascertainment of antibody titers. Studies have used different kinds of ELISA-based assays, other immunoassays, pseudotyped lentivirus assays, and live virus assays for determining these titers.^19^ Additionally, there is variability in the types of antibodies detected by the various assays used in studies. Notably, some studies have used assays that detect the total antibodies generated after booster dose administration, while others have determined neutralizing antibodies through binding assays or live virus assays. Crawford et al use neutralization assays for the estimation of neutralizing antibody.^16^ Jeewandara C et al use ELISA for total antibody, and surrogate virus neutralization test (sVNT) for neutralizing antibody.^8^

A significant finding of this study was that the rise in antibody titer using the pseudotyped lentivirus assay was observed to be lower than that obtained with SARS-CoV-2 IgG II Quant chemiluminescent microparticle immunoassay (CMIA). Although there was a rise in post-booster titer with both assays, it was not statistically significant in the pseudotyped lentivirus assay. One possible reason for this observation could be that analytes differ in the two assays, with CMIA detecting total antibodies and pseudotyped lentivirus antibody neutralization assay detecting neutralizing antibodies. Therefore, one factor contributing to this observation could be that while there is a significant rise of total antibodies even beyond 2 months following the booster dose administration, the neutralizing antibodies seem to decline after this timeframe, as most studies report peaking neutralizing titers 20 days following vaccination.^20^,^21^ However, since neutralizing antibodies are more strongly correlated to actual vaccine efficacy, results from the neutralizing assay may be more representative of the actual immunogenicity following vaccine administration.^22^

In our study, the time period from booster dose to sample collection starts from 2 months, with 23 samples collected from 2-6 months, and 20 samples collected after 6 months. No significant difference in the titers were observed when participants were segregated based on booster dose administration timing. Therefore, the current study remains inconclusive with regard to any differences in titers based on a cut-off time frame of 6 months. This timeframe has been proposed by many studies as the optimal time to receive further shots of vaccines. Similarly, it remains inconclusive with regard to any significance of prior COVID-19 positivity on the change in antibody titers observed after the booster dose administration. Gender also did not seem to impact antibody titers in the current study, though other studies have variably correlated higher antibody titers with genders.^23^,^24^

### Limitations

Some limitations of the study include a small sample size, with varying time intervals between the booster dose and sample collection. However, since samples for both CMIA and the pseudotyped lentvirus antibody neutralization assay were collected at the same point, the study does highlight the differences in results obtained on the same study population with the two assays. The lack of statistical significance of the pre and post-booster titer differences using the assay is likely attributable to a small sample size. Alternatively, it must also be noted that the antibody neutralization assay is a laboratory-developed test, and thus suffers from some limitations inherent in the lack of standardization. Additionally, it is a semi-quantitative test as quantitative controls were unavailable at the time of study was conducted. As of now, WHO has developed the calibrator for pseudo typed lent virus assay. Additionally, although we found no statistically significant differences of titers based on gender, and prior COVID-19 exposure, our limitation of sample size hinders any conclusive evidence on that front. Gender matching, as well as a larger sample size, could bring out some differences in the antibody titers based on gender and prior COVID-19 exposure.

## CONCLUSION

We conclude that a heterologous booster dose with Pfizer–BioNTech COVID-19 vaccine following two doses of BBIBP results in increased total antibody titers. However, long-term neutralizing antibody titers may not show these trends. The latter finding may have implications for the need of revaccination even following a single booster shot. Increasing the power of the study through a larger and more uniform sample size, however, may result in the detection of statistical significance of the rise of neutralizing antibody titers following booster dose administration. It may also bring out differences based on gender, and duration of time following booster dose administration. Notwithstanding, the study does highlight that neutralizing titers may show a steeper time-dependent deceleration than total titers. This also highlights the need for methodological standardization used for reporting these titers.

### Authors’ Contribution

**JA**, conceived, designed, edited, and approved the manuscript & is responsible for the integrity of research. **SH**, did manuscript drafting, experiments, and acquisition of data. **FN**, did experiments and the acquisition of data. **FK**, conceived and designed the study.
